# Therapeutic Potential of Selenium in Glioblastoma

**DOI:** 10.3389/fnins.2021.666679

**Published:** 2021-05-28

**Authors:** Eduard Yakubov, Thomas Eibl, Alexander Hammer, Markus Holtmannspötter, Nicolai Savaskan, Hans-Herbert Steiner

**Affiliations:** ^1^Department of Neurosurgery, Paracelsus Medical University, Nuremberg, Germany; ^2^Department of Neuroradiology, Paracelsus Medical University, Nuremberg, Germany; ^3^Department of Neurosurgery, University Medical School Hospital, Universitätsklinikum Erlangen (UKER), Friedrich–Alexander University Erlangen–Nürnberg (FAU), Erlangen, Germany; ^4^BiMECON Ent., Berlin, Germany

**Keywords:** selenium, glioblastoma, glutamate, SLC7A11, microglia

## Abstract

Little progress has been made in the long-term management of malignant brain tumors, leaving patients with glioblastoma, unfortunately, with a fatal prognosis. Glioblastoma remains the most aggressive primary brain cancer in adults. Similar to other cancers, glioblastoma undergoes a cellular metabolic reprogramming to form an oxidative tumor microenvironment, thereby fostering proliferation, angiogenesis and tumor cell survival. Latest investigations revealed that micronutrients, such as selenium, may have positive effects in glioblastoma treatment, providing promising chances regarding the current limitations in surgical treatment and radiochemotherapy outcomes. Selenium is an essential micronutrient with anti-oxidative and anti-cancer properties. There is additional evidence of Se deficiency in patients suffering from brain malignancies, which increases its importance as a therapeutic option for glioblastoma therapy. It is well known that selenium, through selenoproteins, modulates metabolic pathways and regulates redox homeostasis. Therefore, selenium impacts on the interaction in the tumor microenvironment between tumor cells, tumor-associated cells and immune cells. In this review we take a closer look at the current knowledge about the potential of selenium on glioblastoma, by focusing on brain edema, glioma-related angiogenesis, and cells in tumor microenvironment such as glioma-associated microglia/macrophages.

## Introduction

Glioblastoma is by far the most common occurring malignant primary brain tumor in adults, affecting approximately 15% of all primary brain tumors diagnosed annually in the United States (Ostrom et al., 2019) and 20% in Europe (Wohrer et al., 2009; Darlix et al., 2016). Despite the aggressive multimodality strategy, the current prognosis of patients with glioblastoma (WHO Grade IV) is poor, with a median survival time of only 12–15 months (Wen and Kesari, 2008). The aggressive infiltrative growth of malignant glioma cells and the development of tumor angiogenesis are still therapeutic obstacles (Jain et al., 2007). Both, the complex molecular intra- and inter-tumoral heterogeneity of glioblastoma as well as the evidence of glioma stem cells (GSCs) in tumor microenvironment (TME), make a complete surgical resection impossible (Cheng et al., 2013; Soeda et al., 2015). Thus, tumor recurrence is an expected result after high-grade glioma surgery despite maximal and supramaximal resection.

Furthermore, although cell death of all tumor cells was observed in glioblastoma cell lines treated with certain concentrations of the chemotherapeutic agent temozolomide (TMZ, Temcat^®^, Temodal^®^, or Temodar^®^) *in vitro*, the life expectancy of patients with glioblastoma increases by not more than 2.5 months (Stupp et al., 2005). One of the major reasons for this outcome is that this oral alkylating agent has a limited selectivity toward malignant cells. In other words, the very toxic treatments such as radiation and chemotherapy indiscriminately attack all cells, including healthy cells causing extensive cellular damage and cytotoxicity. This gives rise to recurrence of glioblastoma and promotes development of drug resistance in tumor cells. However, in order to develop novel therapeutic strategies to treat these malignant brain tumors successfully, it is indispensable to have a better understanding of why conventional therapies fail to target malignant cells and often result in tumor relapse.

Selenium (Se) is an important nutritional supplement that is becoming more popular in clinical researches. Se is a key component that can be found in vegetables, soil or meat, but it can also be easily obtained as a dietary supplement without prescription. The initial enthusiasm for supplemental Se intake was based on its anti-oxidative functions. Also, Se has been known for many years to have chemo-preventive functions (Yakubov et al., 2014). The use of Se compounds as a therapeutic agent in malignant tumors was first mentioned by the French surgeon Pierre Delbet at the beginning of the last century, describing the death of patients who received lethal overdoses of sodium selenate (Delbet, 1912). That Se might protect against glioblastoma was first suggested in the late 1980s based on observations of reduced serum concentrations of Se in patients suffering from brain malignancies (Philipov and Tzatchev, 1988). Experimental and observational studies demonstrate that a treatment with Se in non-Se-deficient subjects can not only reduce cancer risk, but it can also regulate several molecular processes in tumorigenesis such as a proliferation, redox homeostasis, angiogenesis, brain edema, and the immune system (Streicher et al., 2004; Carlson et al., 2010; Hall et al., 2013; Carlisle et al., 2020).

In the present review, we summarize the current knowledge about the potential of Se on the treatment of malignant brain tumors. Particularly, we focus on brain edema, glioma-related angiogenesis, and cells in TME such as glioma-associated microglia/macrophages.

## Material Search Strategy

Scopus and Web of Science were the main tools of systematic literature searching, whereas PubMed was used as an additional database. The primary time period for the review was January 2000 until January 2021. Research articles and reviews were identified using the search terms (title, abstract, and keywords) “selenium” or “selenoprotein” either alone or in combination with cysteine, glutamate and glutamine as well as these combined with the terms “glioblastoma,” “glioma,” “cancer,” “stem cell,” “brain edema,” “angiogenesis,” “energy metabolism,” and “microglia.” Additional searches were performed for the exact proteins, namely COX-2, GPx4, GLS, MIF, SLC7A11, and SEPHS2. Relevant papers identified by this search were reviewed, and the references therein were further considered for other useful leads. Epidemiological research on Se supplementation, studies on Se speciation and neurodegenerative disease were not within the main focus of the current review as they have been reviewed recently (Weekley and Harris, 2013; Lopes da Silva et al., 2014; Vinceti et al., 2014, 2018; Yakubov et al., 2014; Solovyev, 2015; Collery, 2018; Gandin et al., 2018).

## Glioma Microenvironment and the Tumor Zone Model

**FIGURE 1:**
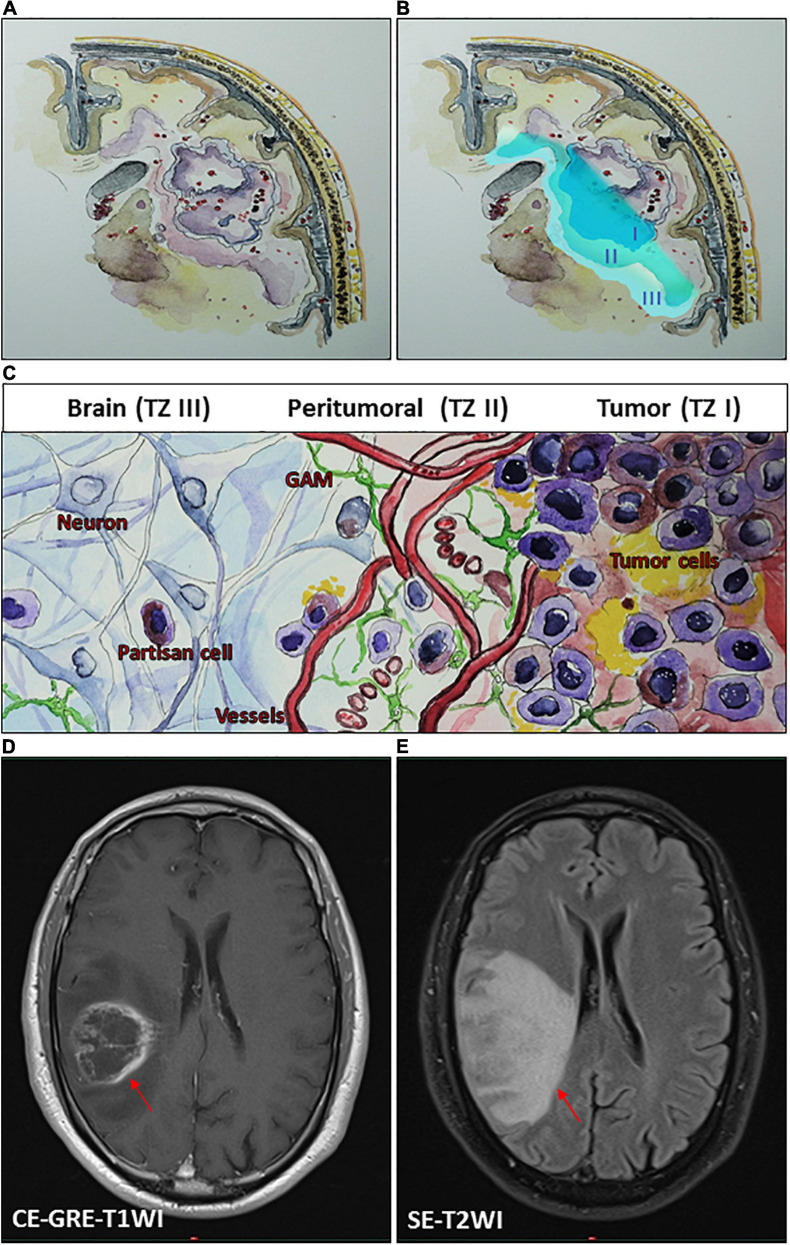
Conceptual schematic illustration of glioma microenvironment classifying the tumor into three heterogeneous tumor zones (TZ I–III). **(A,B)** An illustrative representation of TZ I–III. **(C)** A schematic illustration of glioma microenvironment showing the cellular level of TZ I–III. TZ I comprises the tumor core cells and can be spotted as the contrast-enhancing regions on magnetic resonance imaging (MRI) **(D)**. TZ II, the most biologically active area of the tumor can be observed on MRI as the area of perifocal edema **(E)**, which is characterized by its specific pro-angiogenic microenvironment and the presence of glioma-associated microglia/macrophages (GAM) and transitory cells. TZ III consists mainly of healthy brain tissue and contains isolated partisan cells, which are probably responsible for tumor recurrence following surgery. **(D,E)** Illustrative MRI scans of a patient with a right parietal glioblastoma (WHO grade IV) by using a 1.5 tesla Ingenia scanner (Philips Healthcare, Best, Netherlands). **(D)** A T1-weighted contrast-enhanced gradient-echo imaging (CE-GRE-T1WI), demonstrating the TZ I (arrow). **(E)** A T2-weighted spin-echo imaging (SE-T2WI), showing the TZ II (arrow).

The debate over how much to push the limits of surgical resection for malignant gliomas is not a recent controversy. Therefore, one can conclude that the most favorable outcomes of glioma surgery are achieved in cases with supramaximal resection. Nonetheless, one of the founding fathers of modern neurosurgery, Walter Dandy, performed hemispherectomies in 1928 for patients with malignant gliomas and found that these tumors still recurred on the contralateral side despite such extremely aggressive resection (Dandy, 1928). A rationale for an invasive migration of glioma cells may be explained based on a theoretical consideration of the glioma microenvironment, classifying the tumor into at least three zones (Eyupoglu et al., 2013). The borders of each one of these transition zones may show a smooth shift into the next tumor zone (Figures 1A–C). The main section of the tumor, tumor zone I (TZ I), comprises the tumor core cells and can easily be spotted as the contrast-enhancing regions on magnetic resonance imaging (MRI) (Figure 1D).

Tumor zone II (TZ II) – the peritumoral zone – consists of glioma “transitory cells,” due to the fact that these are cells that show some of the histological characteristics of the glioma core cells that can be found in TZ I. TZ II is rated as the most biologically active area of the tumor because, aside from containing glioma core cells, it also contains glioma-associated microglia and endothelial cells (Figure 1C). This area displays hypervascularization, which also represents a challenge in adjuvant treatments. Although malignant gliomas show an accumulation of immune cells, these cannot generate an adequate immune response. The extension of TZ II can be observed on MRI as the area of perifocal edema (Figure 1E). Tumor zone III (TZ III) contains the so-called “partisan cells” including isolated glioblastoma cells, tumor-initiating (glioma stem) cells, or precursor cells. TZ III, compared to TZ I or TZ II, appears to be clinically silent, which can be challenging for therapy because it mainly consists of brain tissue. However, partisan cells are probably responsible for tumor recurrence following surgery.

Various factors are secreted and released in these zones, triggering tumor expansion and encouraging key mechanisms for tumor cell progression. These factors foster glioblastoma development and can induce tumor angiogenesis, increase the perifocal edema, lead to neural cell death, paralyze immune cells, or stimulate its proliferation and invasion (Figure 1; Savaskan et al., 2008; Yakubov et al., 2014; Choi et al., 2015; Ghoochani et al., 2016a, b). An ideal surgical approach within malignant gliomas implies an exhaustive resection of TZ I with partial resection of TZ II. A complete resection of TZ II can rarely be reached. However, a supramaximal resection of all tumor zones (TZ I–III) is particularly unattainable. Even though further tumor cell reduction can be achieved with adjuvant radiochemotherapy, some persistent partisan cells will inevitably remain within the brain (Robin et al., 2014). In this scenario, surgery and following radiochemotherapy is iterated, primarily aiming to work against the space occupying consequence of the recurrence. This cycle results in a selection of persistent tumor cells leading to acquired chemoresistant tumors. Due to this repetitive selection, the period time from surgery until recurrence diminishes with every next cycle. These cycle scenarios cause that measures such as surgery or radiochemotherapy are not able to control tumor progression onward.

Therefore, neuro-oncological approaches concerning malignant gliomas are essentially challenging. The current limitations in surgical treatment and radiochemotherapy outcomes encourage researches to look up for better suitable treatments that can promise better achievements.

## Glutamate Excitotoxicity and Selenium in Glioblastoma

Aside from uncontrolled proliferation and diffuse brain infiltration, neurodegeneration and brain edema represent the feared hallmarks of malignant brain tumors (Wen and Kesari, 2008; Savaskan and Eyupoglu, 2010). Brain edema crucially contributes to the clinical course and outcome of patients with high-grade gliomas (HGGs, WHO grades III and IV) (Carlson et al., 2007). Glioma-induced brain edema is caused by two interdependent mechanisms: HGGs primarily induce abnormal angiogenesis with impaired blood-brain barrier, allowing plasma to enter the interstinal space referred to as vasogenic edema (Wen et al., 2010). Furthermore, HGGs induce neuronal cell death and neurodegeneration by which cytotoxic brain edema can be formed inducing neurological deficits and intractable seizures (Savaskan et al., 2008; Pace et al., 2013).

**FIGURE 2:**
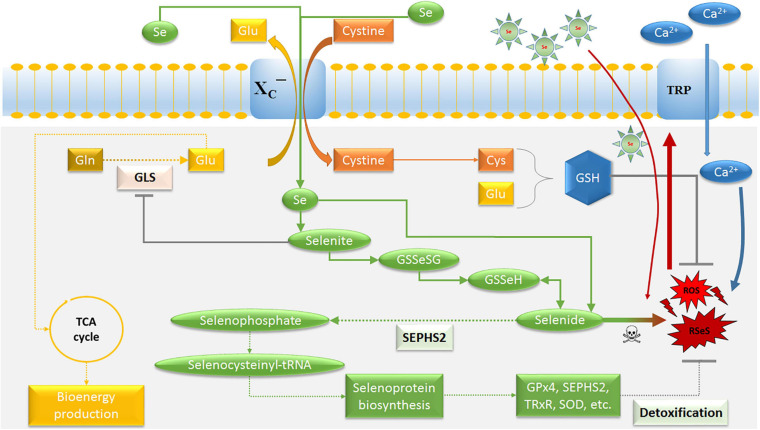
Schematic model for the potential of selenium on glioblastoma cells. The main purpose of the system X_*C*_^−^ transporter is to supply cysteine for the production of the cellular antioxidant GSH. Selenide forms RSeS. Excessive Ca^2+^ influx through Se-induced oxidative stress causes an activation of TRP channels and mitochondrial membrane depolarization, leading to excessive ROS production. Thus, SEPHS2 is essential for survival in glioblastoma cells due to their elevated expression of system X_*C*_^−^ transporter, which induces the import of Se compounds selenite and its conversion to toxic selenide resulting in selenide poisoning and cancer death. The increasing import of Se can be achieved by Se-containing nanoparticles. GLS is a key enzyme for glutaminolysis and bioenergy metabolism, which can be inhibited by selenite. Ca^2+^, calcium; Cys, cysteine; GLS, glutaminase; Glu, glutamate; Gln, glutamine; GSH, glutathione; GSSeSG, selenodiglutathione; GSSeH, selenoglutathione; SEPHS2, selenophosphate synthetase 2; ROS, reactive oxygen species; RSeS, reactive selenium species; Se, selenium compounds; TCA, tricarboxylic acid cycle; GPx4, glutathione peroxidase 4; TRP, transient receptor potential cationic channel; TRxR, thioredoxin reductase; SOD, superoxide dismutase.

The neurotoxic amounts of the amino acid glutamate is thought to play a major role in TME interactions leading to the development of a cytotoxic edema in peritumoral regions (TZ II) (Takano et al., 2001; Savaskan et al., 2008; Marcus et al., 2010; Choi et al., 2015), supporting the role of glutamate in glioma cell infiltration also into the TZ III (Marcus et al., 2010; Corbetta et al., 2019). Nevertheless, glioma stem cells (GSCs) were reported to exhibit high drug and radioresistance with migratory potential, and the enriched proportion of GSCs aggravates the tumor (Bao et al., 2006; Chen et al., 2012; Nusblat et al., 2020). A relevant mechanism is represented by the system X_*C*_^–^ transporter (Figure 2), particularly, of its light chain xCT (SLC7A11). This is instrumental in glioblastoma release of excitotoxic concentrations of glutamate into extracellular milieu, which exchanges intracellular glutamate for extracellular cystine (Choi et al., 2015). Intracellularly, cystine is readily converted to cysteine, the rate-limiting precursor for glutathione (GSH) synthesis, resulting in an increased proliferation and GSH-related drug resistance in various cancers (Doxsee et al., 2007; Narang et al., 2007; Polewski et al., 2017; Guo et al., 2020). Also, there are indications that xCT is implicated in tumor-associated epileptic events and predicts poor survival in patients with glioblastoma (Buckingham et al., 2011; Yuen et al., 2012; Robert et al., 2015). In addition, it was recognized that glutamate/glutamine cycle is a major energy source for tumor cells, including brain tumors (Marin-Valencia et al., 2012; Fendt et al., 2013; Herranz et al., 2015; Tardito et al., 2015). Therefore, targeting the metabolism in GSCs and tumor-associated cells in the TME has recently become one of the most exciting and promising fields for the development of new anticancer treatments (Diwakar et al., 2017).

### Selenium Deficiency in Malignant Brain Tumors

Selenium (Se) is a well-known essential trace element that takes part in many physiological processes, such as aging, immune system function, and anti-oxidant defense with the ability to promote neuronal cell survival (Brauer and Savaskan, 2004; Ray et al., 2006; Reid et al., 2008; Naziroglu et al., 2014; Yakubov et al., 2014; Cardoso et al., 2015). Several studies reported significant low Se levels in patients with high-grade gliomas (HGGs, WHO grades III and IV) (Philipov and Tzatchev, 1988; Al-Rawi et al., 2018; Stojsavljevic et al., 2020). A conventional treatment may even aggravate Se deficiency in patients with HGGs (Zeng et al., 2012) and, consequently, this may promote a negative impact on oxidative stress, immune function, and treatment resistance (Yakubov et al., 2014).

In the past century, it has been observed that reversing Se deficiency in patients with brain tumors improves the condition of patients with neurological side effects such as nausea, unsteady gait, speech disorders, or seizures (Philipov and Tzatchev, 1990; Pakdaman, 1998). These patients were given either 1,000 μg inorganic Se in form of sodium selenite by infusion in physiological saline per day for 4–8 weeks (Pakdaman, 1998), or 150 μg organic Se in combination with 60 IU vitamin E for several weeks to 1 year (Philipov and Tzatchev, 1990). This was also demonstrated in rodent xenograft-glioma model (Hervouet et al., 2013; Yakubov, 2019). Se-excessive diet and intrathecal treatment of Se were associated with a prolonged survival and delayed neurological deficits compared to controls or dietary Se-deficient animals (Yakubov, 2019). Similarly, Hervouet et al. (2013) reported a beneficial neurological effect of a diet mixture of α-tocopherol, β-carotene, Se, vitamin C, and zink. Remarkably, the invasive morphology of malignant cells and the tumor aggressiveness were decreased after treatment of Se in both xenograft models (Hervouet et al., 2013; Yakubov, 2019).

### Selenium Effects in Glioblastoma Cells

The anti-gliomagenic and neuroprotective effect of Se appeared to have a role in regulation of calcium channels, in particular, transient receptor potential (TRP) cationic channels (Figure 2), including TRP melastin 2 (TRPM2) and vanilloid 1 (TRPV1) (Ataizi et al., 2019; Ertilav et al., 2019; Naziroglu et al., 2020; Akyuva et al., 2021). The involvement of TRPM2 channel on glioblastoma progression was recently reported (Alptekin et al., 2015; Ertilav et al., 2019). Interestingly, Naziroglu’s group reported that Se-species stimulate glioblastoma cell death by increasing the mitochondrial ROS generation and the intracellular free calcium concentration through enhanced TRPM2 activation (Ertilav et al., 2019). Nonetheless, protective effects were observed in non-malignant cells such as neurons or microglia cells (Ataizi et al., 2019; Ertilav et al., 2019; Akyuva et al., 2021), suggesting a cellular specificity and higher affinity of Se-species on apoptosis and oxidative stress to glioma cells (Yakubov et al., 2014; Hazane-Puch et al., 2016; Harmanci et al., 2017; Yakubov, 2019). It has been previously reported that low doses of organic and inorganic forms of Se-species have antioxidant properties in malignant cell lines, but there are apoptotic effects with high dose applications (Uguz et al., 2009; Harmanci et al., 2017).

According to Carlisle et al. (2020), a selenium-specific impact on drug-resistant cells selectivity is connected with xCT and selenophosphate synthetase 2 (SEPHS2). SEPHS2 (also known as SPS2) is required for the production of selenoproteins (Figure 2), a group of at least 25 proteins containing selnocysteine residues (Xu et al., 2007), which include antioxidant enzymes such as glutathione peroxidase, thioredoxin reductase, and superoxide dismutase (Papp et al., 2007; Brigelius-Flohe, 2008; Yakubov et al., 2014). Glutathione peroxidase 4 (GPx4) has been implicated in the protection of cancer cells against ferroptosis and chemotherapeutic resistance (Yang et al., 2014; Hangauer et al., 2017). However, GPx4 is also required for the detoxification of Se, in particular of the Se-xCT-SEPHS2 pathway intermediate selenide (Carlisle et al., 2020). Inhalation of hydrogen selenide is reported to be toxic (Schecter et al., 1980). Also, it has been suggested that selenide reacts with water during its decay into elemental Se, forming reactive Se and oxygen species (RSeS/ROS) such as hydroxy radicals, superoxide and hydrogen peroxide (Seko and Imura, 1997; Peyroche et al., 2012), suggesting a potential mechanism for its toxicity (Figure 2). This was confirmed by a report which showed an improved pharmacologically potential of synthetic selenocyanates in glioblastoma cells as compared to the naturally occurring phenylalkyl isothiocyanates (Sharma et al., 2008). Increasing lipophilicity or isosterically replacing sulfur with Se in the structure-activity of the original precursor compounds, enhanced the toxicity of Se toward glioma cells by affecting their cell redox state. Thus, SEPHS2 is essential for survival in GSCs because of its elevated expression of xCT. The overexpression of xCT leads to the import of dietary Se compound selenite and converts it into toxic selenide, resulting in selenide poisoning and cancer death (Carlisle et al., 2020). Due to the fact that normal cells do not considerably express xCT, and do not depend on SEPHS2 detoxification, they remain surprisingly spared. Moreover, the ability of Se to counteract glutamate release is connected with the inhibition of redox-sensitive transcription factors, mainly the nuclear factor kappa B (NF-κB) and hypoxia-inducible factor 1 (HIF-1) (Savaskan et al., 2003), reducing glutamate- and hypoxia-induced ROS production in TZ II and TZ III (Mehta et al., 2012). These aspects confirm the existence of a redox reprogramming in GSCs, which differs from, but is possibly influenced by, the other cellular components in the TME.

### Selenium-Containing Nanotreatment of Glioblastoma

The beneficial and anti-gliomagenic effects of Se depends on its dose and routes of administration (Weekley and Harris, 2013; Yakubov et al., 2014; Rayman, 2020). Having this in mind, the application of nanotechnology enhanced the therapeutic efficiency of Se-species and reduced side effects on normal cells (He et al., 2017; Ferreira et al., 2019; Geoffrion et al., 2020). Nanoparticle delivery systems with Se-carrier were designed to overcome the blood-brain barrier for the selective treatment of HGGs (You et al., 2016; Song et al., 2018). Recently, Jiang et al. (2014) developed polysaccharide from *Gracilaria lemaneiformis* conjugated to Se nanoparticles. The anti-gliomagenic effects in U87 and C6 glioma cell lines were significantly enhanced by recognizing the α_*v*_β_3_ integrin receptor. There is a higher expression of the integrin receptor in U87 cells than C6 cells, which leads to achieve a higher uptake of inorganic Se by U87 cells via receptor-mediated endocytosis, subsequently inducing and enhancing the production of ROS (Figure 2). This leads to glioblastoma cell apoptosis by activating p53 and MAPK cell signal pathways (Jiang et al., 2014). Other laboratory studies confirmed similar results in glioblastoma treatment with some modifications in surface decorating ligands for Se nanoparticles like HER2 and arginylglycylaspartic acid (You et al., 2016; Jardim et al., 2017; Song et al., 2018). Also, it has been demonstrated that Se-containing nanoparticles treatment significantly reduced both the bioenergy metabolism and the invasion of drug-resistant glioma cells (Xu B. et al., 2020), while benign cells remained viable indicating that Se toxicity is selective for glioma cells.

In order to further enhance the therapeutic effect, additional studies have extended the advantages of Se-containing nanomechanisms to deliver chemotherapeutic drugs at a higher concentration. Cheng et al. (2012) demonstrated a superior anti-tumor activity by incorporating Se into temozolomide (TMZ). TMZ-resistant tumor cells could be also effectively be treated with this compound. By comparing TMZ and TMZ-Se, the researchers demonstrated the properties of TMZ-Se as a compound that is able to trigger cell-death more rapidly, with a high apoptosis-inducing activity and as a compound that induces a stronger autophagic response.

### Selenium and Heat Shock Protein in Glioma

Heat shock proteins (HSPs) are highly conserved, ATP-dependent chaperone molecules which are expressed rapidly under stress conditions and form a protective microenvironment necessary for gliomagenesis (See et al., 2011; Jego et al., 2013; Iglesia et al., 2019). Glioma microenvironment condition and notable Se deficiency significantly promote the expression of many HSPs – particularly HSP70 and HSP90 – leading to drug resistance (Alexiou et al., 2014; Beaman et al., 2014; Wu et al., 2016; Zhang et al., 2020a, b). In this respect, most of the studies showed that HSPs are actively involved in glioma-related angiogenesis, energy metabolism, and aggressive glioma by the activation of survival pathway such as PI3K/Akt signaling pathway (Choi et al., 2014; Rajesh et al., 2017, 2019). Interestingly, the treatment of glioblastoma cells by antioxidant Se has been shown to decrease oxidative stress and, as a result, HSP expression could be decreased as well (Zhang et al., 2020a).

Previous reports demonstrated that sodium selenite downregulates histone deacetylase (HDAC) activity in glioblastoma cells in a dose-dependent manner (Hazane-Puch et al., 2016). In consequence, this leads to a caspase-3-dependent apoptosis, cell cycle arrest at the G2 phase, and decreased MMP2 activities. The downstream targets of HDACs, HSP90, is also downregulated in malignant cells. The inhibition of tumor HSPs by Se supplementation results in degradation of oncoproteins such as Akt, RAF-1, and VEGFR (Chan et al., 2006; Fu et al., 2016; Hazane-Puch et al., 2016; Yakubov, 2019). Notably, Se-containing derivates of synthetic HDAC inhibitor SAHA (suberoylanilide hydroxamic acid) were significantly more effective in inducing cytotoxicity in different cancer cells than SAHA alone (Desai et al., 2010b; Karelia et al., 2010; Gowda et al., 2012).

Heat shock proteins have attained a great significance in glioma immunotherapy due to their ability to regulate the M2-like polarization of glioma-associated microglia/macrophages (Zhang et al., 2016). Also, an increased immunogenicity of tumor-associated antigens stimulates both innate and adaptive immunity (Moseley, 2000; Gastpar et al., 2005; Specht et al., 2015). Recent randomized clinical trials of vaccination with autologous tumor-derived HSP-peptide complex have been shown to improve survival in patients with newly diagnosed and recurrent glioblastoma (Crane et al., 2013; Bloch et al., 2014; Ji et al., 2018). Interestingly, vaccine nanoformulations allowed combining Se nanoparticles with siRNA and HSP70, increasing their anticancer activity and selectivity between malignant and healthy cells (Li et al., 2016). The development of innovative administration routes and the advances in creating more efficient and safe carriers with Se-containing derivates opened new doors to treatment possibilities against brain malignancies that needed to be further explored and researched.

## Selenium and Angiogenesis

The influence of TME on glioblastoma cell behavior plays a crucial role leading to diffuse tumor growth and its invasive capacity. The presence of low tumor oxygenation, also known as hypoxia, strongly correlates with glioma invasiveness (Evans et al., 2004). Hypoxia is also a well-known stimulus for angiogenesis (Carmeliet and Jain, 2000; Seidel et al., 2010). The hypoxic niche is enriched by GSCs due to glioma-induced vascular abnormalities and it induces resistance to drugs and radiation in HGGs (Alexandru-Abrams et al., 2014). A fundamental cellular process, which occurs subsequent to hypoxia, is the activation of HIF-1 (Guillemin and Krasnow, 1997).

Importantly, it has been recently shown that Se reduced tumor-related angiogenesis by inhibition of angiogenic factors via the suppression of the PI3K/Akt/HIF-1 signaling pathway (Liu et al., 2016; Yakubov, 2019). It must be emphasized that sodium selenite, even at high concentrations (50 μM), had no influence on the angiogenesis of the healthy brain parenchyma and even had a cytoprotective effect in *ex vivo* glioma-induced brain slices. Furthermore, this inorganic Se compound inhibited the growth of malignant gliomas and reduced the development of tumor-related vascular formation in TZ II (Yakubov, 2019). It could be demonstrated that migration patterns of glioblastoma cells along the peritumorally formed tumor-induced microvascularization was significantly inhibited by selenite in a concentration-dependent manner. There is also evidence that selenite can decrease epidermal growth factor receptor (EGFR) expression, leading to apoptosis and comprehensive alterations in the expression of matrix metalloproteinases (MMPs) (Rooprai et al., 2007). Although most MMPs (except MMP-25) were decreased, it could be seen that their natural inhibitor, tissue inhibitor of metalloproteinase (TIMP), increased. The findings of Yoon et al. (2001) reported as well that Se can contribute to prevent migration of endothelial cells through the extracellular matrix (ECM) and inhibit MMP expression and tumor invasion.

Besides, a growing body of evidence shows that glutamate is able to regulate arteriole diameter, blood-brain barrier disruption and vasodilation (Sharp et al., 2003; Liu et al., 2010; LeMaistre et al., 2012; Fan et al., 2017; Peyton et al., 2018). The dependency of glioblastoma and tumor-associated endothelial cells on glycolysis, but also glutaminolysis for energy production, opens further opportunities to reduce tumor-related angiogenesis (Seyfried et al., 2015; Artzi et al., 2017). Glutamine consumption is often increased in malignant tumors and the inhibition of intracellular glutaminase (GLS) activity – which converts glutamine into glutamate – has been shown to reduce proliferation and angiogenesis of tumor cells of different origin (Draoui et al., 2017; Zhao et al., 2017; Bruntz et al., 2019; Restall et al., 2020). Recent studies have demonstrated that Se-induced inhibition of glutaminolysis in malignant cells resulted from the suppression of GLS activity (Zhao et al., 2017; Bruntz et al., 2019). These findings suggest that selenite inhibits GLS activity leading to a decreased bioenergy metabolism and GSH synthesis in cancer and tumor-associated endothelial cells (Figure 2). As a consequence, a low level of intracellular glutamate prevents endothelial cell proliferation, resulting in impaired tumor-related angiogenesis (Kim et al., 2017), and accelerating selenide-dependent cancer death (Carlisle et al., 2020).

## Selenium and Glioma-Associated Microglia

Microglia are macrophage-like cells that are considered the major immune cells in the brain (Prinz et al., 2017). Most of the immune cells in HGGs consists of microglia/macrophages, which can sometimes equal the number of tumor cells (Morantz et al., 1979; Roggendorf et al., 1996). Also, it has been suggested that neoplastic microglia/macrophages with phagocytic properties, arising through possible fusion hybridization, can comprise an invasive cell subpopulation within glioblastoma (Huysentruyt et al., 2011). Nevertheless, the role of microglia in tumor progression has been shown to be double-edged, as these cells can both promote tumor rejection and stimulate tumor growth depending on their current functional phenotype (Lisi et al., 2014). Two phenotypes have been described as classically activated microglia (M1), considered a pro-inflammatory, and alternatively activated microglia (M2) a pro-angiogenic, immunosuppressive (Ding et al., 2014). However, the validity of this distinction has been debated (Ransohoff, 2016).

Glioma-associated microglia/macrophages (GAMs) are a major component of tumor infiltrates resulting from either resident microglia or monocytes-derived macrophages (MDMs) from the blood (Li and Graeber, 2012; Glass and Synowitz, 2014; Cai et al., 2020). The tumor-supportive M2-polarization of GAMs seemed to predominate in TZ I and TZ II (Ellert-Miklaszewska et al., 2013; Choi et al., 2015; Ghoochani et al., 2016a,b), although anti-tumoral effects of M1-GAMs were also described (Galarneau et al., 2007). However, hypoxia and glutamate-induced excitotoxicity have been found to play an essential regulatory role in immune response modulation (Laoui et al., 2014; Choi et al., 2015; Henze and Mazzone, 2016). The activation of transcription factors, mainly HIF-1 and NF-κB, are pivotal molecular pathways involved in tumor cells (Mantovani et al., 2008; Yakubov, 2019). These transcription factors modulate the production of macrophage migration inhibitory factor (MIF) and cyclooxygenase-2 (COX-2), leading to overexpression of prostaglandin E2 (PGE2). Immune cells, especially microglia/macrophages are recruited and activated through these transcription factors (Ghoochani et al., 2016a), which leads to glutamate and cytokine release, ROS generation, and overload of calcium influx (Dalla Puppa et al., 2007; Socodato et al., 2018; Yildizhan and Naziroglu, 2020). However, NF-κB is highly dysregulated in HGGs and GAMs (Garkavtsev et al., 2004; Nam et al., 2008; Zanotto-Filho et al., 2017).

It has been shown in brain malignancies that microglia/macrophages can lead to a production of angiogenic factors such as VEGF. This is important for tumor progression through different signaling pathways such as HIF-1/ROS and NF-κB (Brandenburg et al., 2016; Schuett et al., 2017; Blank et al., 2020). Interestingly, studies have demonstrated that Se inhibits the activation of NF-κB in microglia/macrophages (Savaskan et al., 2003; Nam et al., 2008; Xu J. et al., 2020). It is plausible that a disturbance of NF-κB in microglia/macrophages by Se supplementation can lead to a decrease of the local immune suppression and may also affect the expression of cell survival factors such as interleukin-6 (IL-6), tumor necrosis factor alpha (TNFα), MIF, COX-2, and PGE2 (Xin et al., 2010; Fujita et al., 2011; Li et al., 2018; Liang et al., 2018; Cheng et al., 2020). Recently, it has been shown that Se is able to suppress the proinflammatory activity, and mitochondrial stress via inhibition of calcium channels such as TRPM2 channel (Akyuva et al., 2021). Additional supplements of selenoproteins were also associated with an increasement of migration and phagocytosis properties of microglial cells (Meng et al., 2019). However, COX-2 appears to be one of the key factors regulated by Se (Desai et al., 2010a). There is evidence that Se-dependent inhibition of COX-2 activity with subsequent PGE2 reduction can affect possible cell fusion hybridization in malignant cells (Filippova and Nabors, 2020), resulting in inhibition of glioblastoma growth *in vitro* and *in vivo* (Joki et al., 2000; Fujita et al., 2011; Altinoz et al., 2018). Moreover, GAMs produce high levels of PGE2 through the expression of COX-2 (Badie et al., 2003). Recently, it has been shown that Se through selenoprotein expression decreases the production of PGE2 in microglia/macrophages (Vunta et al., 2008). PGE2 was shown to affect the expression of programmed cell death ligand 1 (PD-L1). This particular expression is associated with tumor progression in gliomas (Litak et al., 2019).

Although these researches pointed Se as a relevant micronutrient in the treatment of brain malignancies, further investigations on the efficacy of Se TME and glioma-associated microglia cells are required.

## Conclusion

Despite the recent technical improvements in neuro-oncology and oncologic neurosurgery, glioblastoma still remains a lethal medical condition. Se has been independently reported as a promising trace element with anticancer properties. This review provides a consolidated overview of Se potential in glioma microenvironment. On the molecular level, there are evidences that Se operates directly on the redox homeostasis and via selenoprotein regulation. Through its intriguing biology, this trace element holds a center stage in glioblastoma. Se affects bioenergy metabolism, modulates the immunological response, and inhibits tumor angiogenesis. Considering the researches in relation to the potential of Se, it can be concluded that Se represents a promising agent in neuro-oncology.

However, the insights of this review arise further questions in relation to the paradoxical effects of this micronutrient. Challenging for further researchers addressing the detrimental effects of Se could be the fact that Se deficiency in patients with malignant gliomas are more common. Also, it would be worthwhile a further mechanistic glimpse into the role of Se compounds in TME and tumor-associated cells such as GAMs. The creation of innovative Se derivates in nanomedicine approaches provide new therapeutic weapons against glioblastoma and expand the range for new researches. Novel nanomedicine Se-derivates can overcome the low therapeutic range and selectivity of Se and improves the general cytotoxic profile in normal cells.

## Author Contributions

EY designed the structure and contents of the review and wrote the manuscript. EY and TE prepared all figures. All authors provided critical revisions to the article and contributed to the article and agreed to submit the manuscript in its current state.

## Conflict of Interest

NS was employed by the company BiMECON Ent. The remaining authors declare that the research was conducted in the absence of any commercial or financial relationships that could be construed as a potential conflict of interest.
